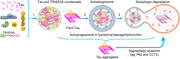# An autophagy adaptor TRIAD3A promotes tau fibrillation by phase separation

**DOI:** 10.1002/alz.095036

**Published:** 2025-01-09

**Authors:** JIECHAO ZHOU

**Affiliations:** ^1^ Solomon H. Snyder Department of Neuroscience, Johns Hopkins University School of Medicine, Baltimore, MD USA

## Abstract

**Background:**

Multiple neurodegenerative diseases are characterized by aberrant proteinaceous accumulations of tau. The maintenance of tau proteostasis reportedly involves the clearance of insoluble tau aggregates by aggrephagy receptors. However, the precise molecular mechanisms underlying both tau fibril formation and degradation remain largely elusive.

**Method:**

We conducted comprehensive investigations, employing both in vivo and in vitro methodologies, to analyze the phase separation of TRIAD3A. Leveraging biochemical assays alongside IHC‐IF and CLEM, identified TRIAD3A as an autophagy adaptor. Employing the TRIAD3A‐TurboID proximity labeling strategy, coupled with CoIP techniques in both in vivo and in vitro settings, we elucidated the binding of TRIAD3A to Tau. Furthermore, employing a tauopathy mouse model, and conducting colocalization studies in human Alzheimer’s disease (AD) brain tissue, we investigated the impact of TRIAD3A on tau homeostasis.

**Result:**

Here, we report an RBR‐type E3 ligase TRIAD3A functions as an autophagy adaptor for tau. TRIAD3A(RNF216) is an essential gene with mutations causing age‐progressive neurodegeneration. Our studies reveal that TRIAD3A E3 ligase catalyzes mixed K11/K63 polyubiquitin chains and self assembles into liquid‐liquid phase separated (LLPS) droplets. Tau is ubiquitinated and accumulates within TRIAD3A LLPS droplets and via LC3 interacting regions targets tau for autophagic degradation. Unexpectedly, tau sequestered within TRIAD3A droplets rapidly converts to fibrillar aggregates without the transitional liquid phase of tau. In vivo studies reveal TRIAD3A decreases the accumulation of phosphorylated tau in a tauopathy mouse model, and disease‐associated mutation of TRIAD3A increases accumulation of phosphorylated tau, exacerbates gliosis, and increases pathological tau spreading. In human Alzheimer’s disease brain, TRIAD3A colocalizes with tau amyloid in multiple histological forms suggesting a role in tau proteostasis.

**Conclusion:**

TRIAD3A is the first autophagic adaptor that utilizes E3‐ligase and LLPS as a mechanism to capture tau, thus preventing pathologic tau aggregation and spread.